# Long-term outcomes of therapist-guided Internet-delivered cognitive behavior therapy for pediatric obsessive-compulsive disorder

**DOI:** 10.1038/s41746-020-00327-x

**Published:** 2020-09-23

**Authors:** Fabian Lenhard, Erik Andersson, David Mataix-Cols, Christian Rück, Kristina Aspvall, Eva Serlachius

**Affiliations:** 1 Centre for Psychiatry Research, Department of Clinical Neuroscience, Karolinska Institutet and Stockholm Health Care Services, 171 77 Region Stockholm, Sweden; 2 Division of Psychology, Department of Clinical Neuroscience, Karolinska Institutet, 171 77 Stockholm, Sweden

**Keywords:** Anxiety, Health services

## Abstract

Cognitive behavior therapy (CBT) is the recommended first-line intervention for children and adolescents with obsessive-compulsive disorder (OCD), but is not broadly accessible. Internet-delivered CBT (ICBT) with minimal therapist support is efficacious and cost-effective, at least in the short term. Whether the therapeutic gains of ICBT for OCD are sustained in the long run is unknown. In this study, 61 adolescents with OCD who participated in a randomized trial of ICBT were followed-up 3 and 12 months after treatment. The proportion of treatment responders and remitters remained stable from post-treatment to 3-month follow-up and increased significantly from 3-month to 12-month follow-up. This study suggests that the gains of ICBT for youth with OCD are not only maintained long-term, but that further improvements continue to occur during follow-up.

## Introduction

Obsessive compulsive disorder (OCD) affects about 2% of children and adolescents^1^ and is associated with significant individual suffering and everyday life impairment^2,3^. Exposure-based cognitive behavior therapy (CBT) is the recommended first-line intervention for childhood OCD^4,5^. Roughly 70% of patients that receive CBT respond to the treatment, defined as a significant decrease of symptoms, and about half of patients remit, i.e., no longer fulfill diagnostic criteria for OCD^4^. Long-term follow-ups of CBT show that these beneficial outcomes are maintained up to three years after treatment^6,7^. However, CBT is not available to all patients, and to overcome existing treatment barriers of e.g., shortage of trained therapists, limited resources and geographical distances, internet-delivered CBT (ICBT) has been developed. ICBT mimics the course and content of standard face-to-face CBT, but is presented via a secure internet portal, very similar to an e-learning online course. “Unguided” ICBT is a form of pure self-help delivered online, whereas in “guided” ICBT the patient receives support from a dedicated therapist via e-mail and/or telephone^8^.

ICBT has extensively been evaluated for adults with OCD^9,10^ with large effect sizes at post-treatment, which were also maintained up to two years after treatment^11^. In young people with OCD, ICBT has been evaluated in three open trials^12–14^, a clinical implementation study across 3 clinics^15^, and one waitlist randomized controlled trial (RCT)^16^. Collectively, results from these studies consistently show feasibility and acceptability of ICBT, as well as large within-group, and moderate between-group, effect sizes. Thus far, 3-month and 6-month follow-up results from these studies are available, indicating that the gains are not only maintained but patients tend to continue improving further during the follow-up^12,13^. This is normally not to be expected in standard face-to-face CBT, where the treatment effects obtained at post-treatment tend to be maintained without changes occurring during the follow-up^6,17^. Before ICBT for youth with OCD can be recommended for implementation in routine clinical care, it would be important to demonstrate that the therapeutic gains of ICBT are maintained long-term. Given the paucity of long-term follow-up data from patients treated with ICBT, in this study we report the naturalistic one-year follow-up data from the participants in the Lenhard et al. (2017) RCT^16^.

## RESULTS

### Sample characteristics

**Fig. 1:**
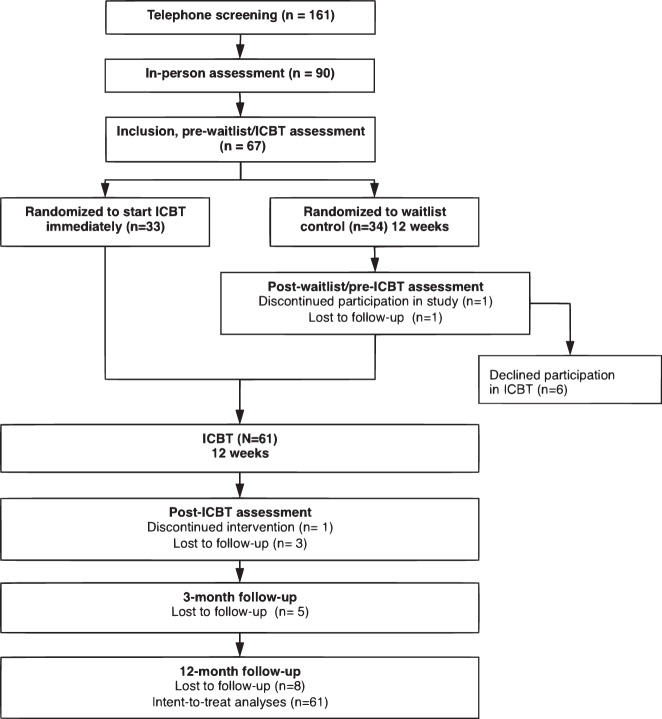
Study flow chart. Flow chart of patient selection, intervention and assessment time points.

The patient study flow chart is presented in Fig. 1. The mean age of the patients was 14.44 (SD = 1.68) years and 43% (*n* = 26) were girls. A minority of patients, *n* = 11 (18%), were on stable selective serotonin reuptake inhibitor (SSRI) medication when entering the study and maintained that medication during the trial. Fourteen patients (23%) had received previous CBT for OCD, defined as at least 5 sessions of exposure and response prevention. For a detailed presentation of the separate results of the two randomized groups at pre-, post-ICBT and 3-month follow-up, see Lenhard et al. (2017)^16^.

### Primary outcome

**Fig. 2:**
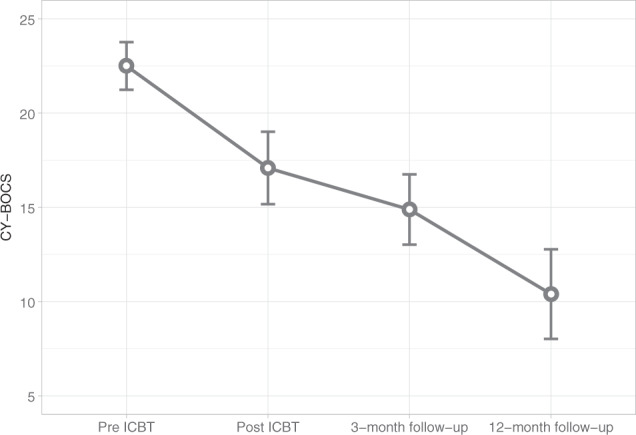
Primary outcome results. CY-BOCS mean scores and 95% confidence intervals at pre-treatment, post-treatment and follow-up (*N* = 61).

Fifty-three (87%) of all participants that received ICBT completed the 12-month follow-up assessment. There were significant mean score changes on the CY-BOCS from pre-ICBT to post-ICBT (*β* = 5.90, *t* = 7.10, *p* < .0001), from post-ICBT to 3-month follow-up (*β* = 2.58, *t* = 4.17, *p* < .001) and additional changes from 3-month to 12-month follow-ups (*β* = 3.99, *t* = 4.92, *p* < .0001) (Fig. 2). Post-hoc analyses did not show any significant effect of ongoing SSRI treatment on CY-BOCS scores over all time points (*β* = −0.02, *t* = −0.03, *p* = .98).

Within-group CY-BOCS effect sizes between the different follow-up time points were in the large range (*d* = 1.10 – 2.15), as were the CGI-S effect sizes (Table 1). CGI-I effect sizes were small from post-ICBT to 3-month follow-up, and in the large range from 3-month to 12-month follow-up (Table 1).

**Table 1:** Outcomes of ICBT from pre-ICBT to 12-month follow-up.

Measures	Pre-ICBT	Post-ICBT	3-month follow-up	12-month follow-up
CY-BOCS				
M (SD)	21.97 (4.20)	16.12 (6.37)	13.48 (6.32)	9.36 (7.34)
Effect size (Cohen’s d^a^)		1.10	1.60	2.15
CGI-S				
M (SD)	4.31 (0.73)	3.38 (1.24)	3.00 (1.38)	2.19 (1.30)
Effect size (Cohen’s d^a^)		0.91	1.20	2.04
CGI-I				
M (SD)		2.78 (1.04)	2.50 (1.01)	1.90 (1.05)
Effect size (Cohen’s d^b^)			−0.27	−0.83
Responders (%)		33%	39%	73%
Remitters (%)		9%	18%	40%

*CY-BOCS* Children’s Yale-Brown Obsessive Compulsive Scale, *CGI-S* clinical global impression-severity, *CGI-I* clinical global impression-improvement.

^a^Cohen’s *d* is based on mean differences between pre-ICBT and the respective post-ICBT or follow-up assessment.

^b^Cohen’s *d* is based on mean differences between post-ICBT and the respective follow-up assessment.

### Treatment response/remission

There was no significant change in the proportion of treatment responders from post-ICBT to 3-month follow-up. In contrast, there was a significant change in proportion of responders from 3-month (39% responders) to 12-month (73% responders) follow-up (*χ*^2^(1) = 12.5, *p* < .001). Similarly, regarding remitter status, there was no significant change from post-ICBT to 3-month follow-up, but the 3-month to 12-month follow-up comparison showed a significant increase in the proportion of remitters (18 to 40%: *χ*^2^(1) = 8.64, *p* < .01). Regression analyses demonstrated that responder status at post-ICBT predicted responder status at both the 3-month follow-up (z = 3.96, *p* < .001) and the 12-month follow-up (*z* = 2.13, *p* < .05). Similarly, remitter status at post-ICBT predicted remitter status at the 3-month follow-up (*z* = *3*.45, *p* < .001) and the 12-month follow-up (*z* = 2.05, *p* < .05).

### Additional treatments during follow-up

Twelve patients (25%) had received additional face-to-face CBT between the 3-month and the 12-month follow-up. Of those, *n* = 10 were classified as non-responders at the 3-month follow-up, meaning that 10 of 30 non-responders had received additional face-to-face CBT before the 12-month follow-up. A total of *n* = 4 patients received SSRIs between the 3-month and 12-month follow-up, all of whom were classified as non-responders at the 3-month follow-up. Three of the patients that had received SSRIs had also received face-to-face CBT. A post-hoc analysis of the non-responders at 3-month follow-up (*n* = 30) revealed that there was no significant mean difference in symptom severity change dependent on whether those patients had received additional evidence-based treatment (face-to-face CBT or SSRIs) or not (*β* = −1.00, *t* = −0.45, *p* = .65). If the patients that had received additional CBT and/or SSRIs were excluded from the main analyses, the pre-ICBT, post-ICBT, 3-month and 12-month follow-up CY-BOCS mean estimates were *M* = 21.7, 14.18, 12.08, and 8.46, respectively. The mean symptom decreases were significant at every time point (*p* < .01).

## Discussion

This study presented the long-term naturalistic follow-up of ICBT for youth with OCD and results indicate not only sustained treatment effects, but also further improvements, both regarding symptom severity, as well as proportions of treatment responders and remitters. Surprisingly, treatment gains appeared to follow a continued linear decrease of symptom severity over time, including the 3-month to 12-month period, and an unexpectedly large increase in the proportion of treatment responders and remitters was observed at the 12-month follow-up. Treatment response and remission at post-ICBT were predictive of treatment response and remission, respectively, at the later time points.

The follow-up period was naturalistic and patients were actively offered additional evidence-based treatment if they were classified as non-responders three months after ICBT. This was done according to the study routines with the aim to provide best available care to those patients who did not respond sufficiently to ICBT. However, there was no statistical significant effect of additional face-to-face CBT during the follow-up, and when patients who had received additional CBT or SSRIs were excluded from the analyses, we still found a similar pattern of continued improvements during the follow-up period. This delayed treatment effect is somewhat unexpected and atypical in regular face-to-face CBT for pediatric OCD^6,17^ or ICBT for adults with OCD^11^. However, a one-year follow-up of children who had received ICBT for anxiety disorders found a similar delayed treatment effect, with significant symptom severity improvements up to 12-months after treatment and a significant increase from 23 to 55% remitted patients from post-treatment to 3-month follow-up and another marked increase to 73% remitters at the 12-month follow-up^18^.

Although tentative, these results indicate that the treatment effect of ICBT for pediatric OCD may accrue in an atypical manner over a longer period of time. If confirmed, these results have important implications both for researchers and clinicians. For researchers, the findings of this and previous ICBT trials^13,16,18^, suggest that as additional gains are expected after the end of treatment, the primary endpoints of clinical trials may need to be pushed forward in time in order to fully capture the treatment effects. Another implication for clinical trials is that sufficient time should be allowed before ICBT is deemed to be unsuccessful and additional treatment forms are offered. Importantly, these findings also pose new questions about the therapeutic mechanisms of behavior change in ICBT compared to traditional CBT. What explains the delayed and progressive effects of ICBT? One obvious difference is the direct presence of the therapist in CBT versus the more distant role of the therapist in guided ICBT. In ICBT, the therapist cannot directly model exposure exercises or encourage patients to quickly reach the top of their exposure hierarchies, all of which could result in slower progress. On the other hand, in ICBT the materials remain online for the duration of the trial and follow-up, thus enabling families to follow the treatment program at their own pace. The latter may result in the steady symptom decrease that we observed over time. Future research should investigate whether there are qualitative differences in the behavior change mechanisms of CBT and ICBT, in order to better understand and improve the outcomes of ICBT. Contributing to the question when and whom ICBT works for, Lenhard et al.^19^ analyzed the material of the current trial and conducted machine learning predictions of the 3-month follow-up treatment responder status based on baseline patient characteristics. In addition, a classic multivariate regression analysis was conducted. The machine learning algorithms were able to predict response to ICBT with good to excellent accuracy, while the regression analysis failed to do so. These results suggest that novel statistical approaches such as machine learning could be useful when exploring the question of patient selection for ICBT.

This study is limited first and foremost by its naturalistic design and additional treatments that were received during the follow-up period. The naturalistic design prohibits claims of causality between ICBT and the 3-month and 12-month outcomes. Although ethically and practically challenging, future studies should aim for longer controlled follow-ups. This may not be feasible when randomizing patients to wait list conditions or other passive control conditions; however, it may be possible when two or more active interventions are evaluated. The study results are also limited by the age range of the included participants, which should be expanded to also include children younger than 12. As a child version of BIP OCD has been developed^12^, this would be possible in future trials. Regarding the assessments, the 12-month follow-up CY-BOCS ratings were conducted via the telephone. Although all raters were experienced and trained in in-person, as well as telephone assessments, thus ensuring similar standards independent of assessment format, this could potentially have introduced a bias. Moreover, the study was conducted in Sweden within a clinical academic environment. To improve the generalizability of the findings, future studies should be carried out in different cultural and routine clinical settings.

This study indicates that the therapeutic gains of ICBT for pediatric OCD are not only maintained over time but that further improvements can be expected up to one year after the end of treatment. Well-controlled replications are warranted, as well as studies that explore the causes of the observed delayed treatment effect of ICBT.

## Methods

### Study design

The study was a follow-up of a previously published randomized controlled trial of a therapist-guided ICBT intervention for adolescents between 12 and 17 years old with OCD^16^. The study was approved by the Regional Ethical Review Board in Stockholm (2016/673-21/2) and registered at ClinialTrials.gov (NCT02191631). Participants were *N* = 67 adolescents with OCD, randomized to either ICBT for 12 weeks (*n* = 33) or a waitlist control of equal length (*n* = 34). After 12 weeks, most participants on the waitlist crossed over to receive ICBT for 12 weeks (*n* = 28). The remaining participants (*n* = 5) declined to receive ICBT after waitlist but agreed to remain in the study and provide follow-up data. All participants were followed-up 3 and 12 months after ICBT. For the current long-term follow-up evaluation, all patients that received ICBT (either initially or after waitlist) were pooled into one group of *N* = 61 treated individuals. There were no statistical differences between the two groups at pre-ICBT regarding symptom severity.

### Participants

Inclusion criteria were: (a) a primary diagnosis of OCD as defined by DSM-5^20^, (b) a total score of ≥16 on the Children’s Yale-Brown Obsessive-Compulsive Scale, CY-BOCS^21^, (c) age between 12 and 17 years, (d) ability to read and write Swedish, (e) daily access to the internet, (f) one parent that was able to co-participate in the treatment, (g) for patients on psychotropic medication: a stable dose for the last 6 weeks prior to baseline assessment. Exclusion criteria were: (a) diagnosed autism spectrum disorder, psychosis, bipolar disorder, or severe eating disorder, (b) suicidal ideation, (c) ongoing substance dependence, (d) not able to read or understand the basics of the ICBT material, (e) completed CBT for OCD within last 12 months (defined as at least 5 sessions of CBT including exposure and response prevention), (f) ongoing psychological treatment for OCD or another anxiety disorder.

### Procedures

Patients were recruited via newspaper advertisements, social media and the local child and adolescent mental health service in Stockholm, Sweden. At a first stage, interested participants were screened via the telephone to broadly assess presence of any exclusion criteria. Thereafter the adolescent was invited to an in-person clinical interview at our unit together with the primary caregivers. During this visit, a comprehensive clinical evaluation was conducted by a trained psychologist, consisting of the semi-structured clinical interview Mini International Neuropsychiatric Interview, MINI KID^22^, and the CY-BOCS^21^. Families fulfilling inclusion criteria were offered participation in the trial and provided with verbal and written information regarding the research project. All included patients provided a signed informed consent form. The CY-BOCS was administered in-person at post-treatment and the 3-month follow-up, and via the telephone at the 12-month follow-up. At inclusion, the main responsible parent was assigned, which was the parent or other caregiver who would be available for assessments at all time points. However, both parents were invited to actively partake in the treatment. The main responsible parent and the child were interviewed at all time points. All assessments were conducted by trained psychologists. If patients were classified as non-responders at the 3-month follow-up, they were offered a referral to a specialized OCD unit for children and adolescents in Stockholm for standard psychological and pharmacological treatment.

### Intervention

The ICBT intervention, called “BIP OCD”, is delivered via a secure internet portal, giving access to 12 chapters of treatment content presented over 12 weeks. The chapters contain material developed from standard evidence-based CBT treatment manuals, and consist of psychoeducation, exposure and response prevention (ERP), cognitive strategies and relapse prevention, with a main focus on ERP. The child’s parents/legal guardians access parent-directed content via a separate account and are provided with five chapters, covering topics such as family accommodation, coping strategies and facilitation of exposure exercises. The family is supported by a licensed psychologist throughout the treatment via written comments on homework exercises, e-mail messages and occasional telephone calls. More detailed information about BIP OCD is presented in the original publication^16^.

### Measures

The primary outcome measure was the CY-BOCS^21^, which is a semi-structured, clinician-rated interview of OCD symptom severity. Symptom severity is rated on ten 4-point Likert-scale items, resulting in a scale range from 0 to 40. A total score of 16 of above is usually used as a cut-off for moderately severe OCD and an indication for treatment.

Secondary outcome measures were the Clinical Global Impression-Severity (CGI-S) and the Clinical Global Impression-Improvement (CGI-I), which are widely used clinician-rated measures to assess global functioning^23^. Both measures are rated on a 7-point Likert-scale item each, with lower ratings indicating less severity (CGI-S) or more improvement (CGI-I).

### Statistical analyses

Descriptive statistics were analyzed and presented as mean values and standard deviations or frequencies and percentages. Symptom severity change was analyzed using mixed effects regression analysis with time as fixed effect and individuals varying over time as a random effect (random slope). Mixed effects regression analysis is also an appropriate method to handle missing data^24^. Multiple comparisons were p-value adjusted using the Tukey method. Effect sizes were calculated as Cohen’s *d* with mean differences divided by the pooled standard deviation. Responder and remitter status was defined according to international consensus^25^, with responder status as a CY-BOCS score symptom severity decrease of at least 35% and a CGI-I rating of 1–“very much improved” or 2–“much improved”, and remitter status defined as a CY-BOCS score of 12 or below, as well as a CGI-S rating of 1–“normal, not at all ill” or 2–“borderline mentally ill”. Paired binary data were analyzed with McNemar tests. All statistical tests were two-sided. All analyses were performed in R^26^.

### Reporting summary

Further information on research design is available in the Nature Research Reporting Summary linked to this article.

## Supplementary information

Reporting Summary

## Data Availability

Data are not available due to European data regulation restrictions in accordance with the General Data Protection Regulation (GDPR).

## Code availability

The statistical code underlying the results in this publication can be provided by the corresponding author upon request.
