# Supplementary material for: Long-term outcomes of therapist-guided Internet-delivered cognitive behavior therapy for pediatric obsessive-compulsive disorder
Source: NPJ Digit Med. 2020 Sep 23;3:124. doi: 10.1038/s41746-020-00327-x (PMC7511358; doi:10.1038/s41746-020-00327-x)
Supplement: Supplementary file 1 — Reporting Summary [file 41746_2020_327_MOESM1_ESM.pdf]

nature research

Corresponding author(s): Fabian Lenhard

Last updated by author(s): Aug 19, 2020

Reporting Summary

Nature Research wishes to improve the reproducibility of the work that we publish. This form provides structure for consistency and transparency in reporting. For further information on Nature Research policies, see our Editorial Policies and the Editorial Policy Checklist.

Statistics

For all statistical analyses, confirm that the following items are present in the figure legend, table legend, main text, or Methods section.

n/a	Confirmed	
☐	☒	The exact sample size (n) for each experimental group/condition, given as a discrete number and unit of measurement
☐	☒	A statement on whether measurements were taken from distinct samples or whether the same sample was measured repeatedly
☐	☒	The statistical test(s) used AND whether they are one- or two-sided<br>*Only common tests should be described solely by name; describe more complex techniques in the Methods section.*
☐	☒	A description of all covariates tested
☐	☒	A description of any assumptions or corrections, such as tests of normality and adjustment for multiple comparisons
☐	☒	A full description of the statistical parameters including central tendency (e.g. means) or other basic estimates (e.g. regression coefficient) AND variation (e.g. standard deviation) or associated estimates of uncertainty (e.g. confidence intervals)
☐	☒	For null hypothesis testing, the test statistic (e.g. F, t, r) with confidence intervals, effect sizes, degrees of freedom and P value noted<br>*Give P values as exact values whenever suitable.*
☒	☐	For Bayesian analysis, information on the choice of priors and Markov chain Monte Carlo settings
☒	☐	For hierarchical and complex designs, identification of the appropriate level for tests and full reporting of outcomes
☐	☒	Estimates of effect sizes (e.g. Cohen's d, Pearson's r), indicating how they were calculated

Our web collection on statistics for biologists contains articles on many of the points above.

Software and code

Policy information about availability of computer code

Data collection	No software was used to collect the data.
Data analysis	R Core Team. R: A Language and Environment for Statistical Computing. R Foundation for Statistical Computing Vienna Austria (2017). doi:10.1038/sj.hdy.6800737

For manuscripts utilizing custom algorithms or software that are central to the research but not yet described in published literature, software must be made available to editors and reviewers. We strongly encourage code deposition in a community repository (e.g. GitHub). See the Nature Research guidelines for submitting code & software for further information.

Data

Policy information about availability of data

All manuscripts must include a data availability statement. This statement should provide the following information, where applicable:

- Accession codes, unique identifiers, or web links for publicly available datasets
- A list of figures that have associated raw data
- A description of any restrictions on data availability

Data are not publicly available due to European data regulation restrictions in accordance with the General Data Protection Regulation (GDPR).

Field-specific reporting

Please select the one below that is the best fit for your research. If you are not sure, read the appropriate sections before making your selection.

☐ Life sciences ☒ Behavioural & social sciences ☐ Ecological, evolutionary & environmental sciences

For a reference copy of the document with all sections, see nature.com/documents/nr-reporting-summary-flat.pdf

Behavioural & social sciences study design

All studies must disclose on these points even when the disclosure is negative.

Study description	Quantitative clinical trial
Research sample	Youth 13 - 17 years of age with obsessive compulsive disorder
Sampling strategy	self- and clinical-referrals, sampling according to inclusion and exclusion criteria presented in the manuscript. Sample size was determined based on previous trials in the field and according to at least 80% statistical power and alpha level of .05.
Data collection	Main outcome was the Children's Yale-Brown Obsessive Compulsive Scale, a semi-structured clinical interview conducted by trained clinicians in-person with the child and primary caregiver. The researcher was not blind to the experimental condition.
Timing	August 2014 through June 2015
Data exclusions	All data was used in the analyses, according to the intent to treat principle.
Non-participation	6 participants declined participation during the trial and 8 were lost to follow-up.
Randomization	Patients were not randomized in this study

Reporting for specific materials, systems and methods

We require information from authors about some types of materials, experimental systems and methods used in many studies. Here, indicate whether each material, system or method listed is relevant to your study. If you are not sure if a list item applies to your research, read the appropriate section before selecting a response.

Materials & experimental systems

n/a	Involved in the study	
☒	☐	Antibodies
☒	☐	Eukaryotic cell lines
☒	☐	Palaeontology and archaeology
☒	☐	Animals and other organisms
☐	☒	Human research participants
☐	☒	Clinical data
☒	☐	Dual use research of concern

Methods

n/a	Involved in the study	
☒	☐	ChIP-seq
☒	☐	Flow cytometry
☒	☐	MRI-based neuroimaging

Human research participants

Policy information about studies involving human research participants

Population characteristics	The mean age of the patients was 14.44 (SD = 1.68) years and 43% (n = 26) were girls. All participants fulfilled diagostic criteria for obsessive compulsive disorder.
Recruitment	Patients were recruited by self-referrals or clinical-referrals. This selection limits the results to self-identified or clinician-identified cases.
Ethics oversight	Regional Ethics Board in Stockholm, Sweden.

Note that full information on the approval of the study protocol must also be provided in the manuscript.

Clinical data

Policy information about clinical studies

All manuscripts should comply with the ICMJE guidelines for publication of clinical research and a completed CONSORT checklist must be included with all submissions.

Clinical trial registration	NCT02191631
Study protocol	Full study protocol can be obtained by the corresponding author.
Data collection	Child and Adolescent Psychiatry Research Center, Child and Adolescent Psychiatry in Region Stockholm, Sweden, August 2014 through June 2015.
Outcomes	Primary outcome was the Children's Yale-Brown Obsessive Compulsive Disorder Scale, which is the gold standard measure in the field, assessed as in-person interview by trained psychologists. Remitter and treatment response was defined according to consensus in the field, see description in the manuscript.
